# “You Have to Make It Normal, That’s What We Do”: Construction Managers’ Experiences of Help-Offering

**DOI:** 10.3390/ijerph22040581

**Published:** 2025-04-08

**Authors:** Emilie Roche, Shane O’Donnell, Noel Richardson

**Affiliations:** The National Centre for Men’s Health, Department of Health and Sports Sciences, South East Technological University, Kilkenny Road Campus, Kilkenny Road, R93 V960 Carlow, Ireland; emilie.roche@postgrad.setu.ie (E.R.); shane.odonnell@setu.ie (S.O.)

**Keywords:** suicide prevention, male-dominated industries, workplace health promotion

## Abstract

Men have a higher risk of suicide compared to women, with those in male-dominated industries such as construction being particularly vulnerable. These industries are typically characterised as ‘macho’ environments, endorsing traditional masculine norms that reinforce mental health stigma and delay help-seeking. The workplace is a promising setting for engaging men with issues around their mental health. Although managers can play a key role by connecting workers in distress with mental health supports, research exploring their experiences of this is limited. This study sought to address this gap by exploring the barriers to and the facilitators of help-offering behaviour among managers in the construction industry in Ireland. Five focus groups were conducted with construction industry managers (*n* = 33) to explore their perspectives on supporting and offering help to workers in distress. Reflexive thematic analysis was used to analyse the data. The findings indicated three key themes: (1) creating the right environment with sub-themes focused on fostering an open culture around mental health on-site and overcoming systemic challenges; (2) navigating the practice of help-offering; and (3) using the right tools for the job. The findings highlight the need for manager training tailored to the construction industry as part of a multi-faceted approach to help-offering within the industry.

## 1. Introduction

Suicide remains a leading cause of death worldwide [1]. The global evidence indicates that in Western countries, men are from two to three times more likely to die by suicide than women [1]. Despite this, the reported rates of suicidal ideation, suicide attempts, and common mental health disorders are consistently higher among women than in men [2,3]. This ‘gender paradox’ regarding suicide is not necessarily indicative of better mental health outcomes among men, but rather highlights the stark differences in suicidal behaviour between the sexes [4]. While men’s use of more lethal means to engage in a suicide attempt is a contributary factor [5], the Social–Ecological Model of health [6] emphasizes that a range of individual (i.e., personal skills and mental health literacy), social (community norms), and environmental (contextual influences) factors shape the male suicide risk. A situational approach to suicide prevention underscores the need to focus beyond the individual and consider the sociocultural influences on suicidal behaviour [7] and the contexts in which individuals live and work. This has led to increased men’s health scholarship on the impact of work environments and the broader cultural and societal influences on men’s mental health.

Work is a key determinant of mental health, having the potential to act as either a protective or risk factor [8]. While work can be a source of meaning that promotes positive mental health for many men [9], harmful psychosocial working conditions can contribute to poor mental health or exacerbate existing mental health conditions [8]. Hazardous working conditions, such as a poor work schedule (e.g., shift work and long working hours), poor organisational culture, role ambiguity or conflict, unsupportive relationships, and low levels of organisational support, can negatively impact a person’s mental health [8]. More specifically, psychosocial risks, such as a high job demand and a low level of job control, are predictors of suicidal ideation among workers [10], while a high level of job stress is associated with suicidal ideation among men in particular [11]. Workers in male-dominated industries—typically defined as more than 70% male employees in the construction, mining, manufacturing, agriculture, utility, transport, and information technology sectors [12,13,14]—may be particularly vulnerable to these risks. The workers in these industries have a higher risk of suicide than the general male population [15,16] and are frequently exposed to risks such as long working hours and financial instability, which have been previously identified as the key drivers of poor mental health [17]. Therefore, efforts to explore the challenges to help-seeking and help-offering behaviours in these industries must account for the wider environmental influences on mental health.

These challenges are further compounded by the ‘macho’ setting of the construction industry. Connell’s relational theory of masculinities [18] provides a useful framework to explore the impact of more traditional masculine norms on help-seeking and help-offering behaviours in these industries. Employees in these industries are reportedly more likely to endorse traditional masculine norms, such as emotional suppression, stoicism, and self-reliance, when compared to those in other sectors [19]. These norms have been linked to greater mental health stigma and delayed help-seeking [19,20,21,22] and are often reinforced in male-dominated industries. Therefore, it is crucial to consider how such norms impact help-seeking and help-offering behaviour in these workplace settings.

Work is a key setting for health promotion, involving collaborative efforts between employers, employees, and the wider society to promote the wellbeing of staff [23]. Such efforts range from individual-level interventions (e.g., skill development) to broader, organisational-level interventions (e.g., policy and environmental changes to better support employees’ mental health). While intervention studies have shown promising findings in male-dominated industries—demonstrating increases in help-seeking, help-offering, reduced stigma, and improved suicide literacy [12,24]—no research to date has specifically focused on how managers navigate help-offering in these industries. Given their critical role in workplace mental health promotion, managers—defined in this study as anyone with a responsibility for the wellbeing of workers—are a priority target group for workplace mental health interventions [25]. Emerging guidelines for workplace mental health place a particular emphasis on training managers to recognise and respond to workers in distress [8,26]. Gatekeeper training for managers is particularly crucial in male-dominated industries, where mental health stigma and the fear of being seen as unreliable often inhibit or delay the disclosure of mental health issues [27].

Managers are considered a key group to train as gatekeepers as they are well positioned to notice the signs of distress in employees and to connect them with mental health support [28]. They can also provide appropriate accommodations to employees in distress [29] and can address the wider psychosocial risks to improve employees’ mental health [30]. More broadly, they can act as role models to help foster a positive culture around mental health and challenge restrictive masculine norms [31]. By openly prompting or endorsing conversations about mental health, they can work towards counteracting the influence of a ‘macho’ culture [32]. Male-dominated industries serve as a unique setting to engage men with issues around their mental health [33], particularly those who might be considered ‘hard to reach’ or less likely to seek help. Strength-based approaches that acknowledge and reframe traditional masculine norms have shown promise in enhancing the acceptability of mental health initiatives in male-dominated industries [34]. For instance, programmes like Mates in Construction have demonstrated the impact of multi-level, peer-support interventions in the construction industry [35,36]. These findings highlight that employees in male-dominated industries are more likely to seek help from informal sources (i.e., peers and colleagues) rather than formal sources (i.e., general practitioners and psychologists) [24]. This underscores the importance of workplace interventions that help normalise conversations around mental health [37], equip the key stakeholders in the workplace to engage with and offer help to a colleague in distress (hereafter referred to as ‘help-offering’), and account for the role of masculine norms in shaping health behaviours.

Despite the very favourable evidence in support of their role in workplace mental health, managers’ perspectives on the acceptability of such a gatekeeper role, including the nuances of the engagement process and the perceived challenges associated with supporting employees’ mental health, remain underexplored. While male help-seeking has been widely researched, there is less emphasis placed on the facilitators or enablers of help-offering behaviour. While some studies have reported increases in the intention to offer help post-intervention in male-dominated industries [24,36], no research to date has qualitatively explored managers’ experiences of engaging with and supporting workers in distress outside the context of intervention evaluation. Understanding the barriers and facilitators that influence managers’ capacity or willingness to offer help to construction workers in distress is crucial for designing effective manager mental health training programmes tailored to this group. While previous studies have explored employees’ perspectives on disclosing mental health issues at work [27,28,37], there has been much less of a focus on understanding the complexities, the motivations, and the sociocultural influences on help-offering behaviour in the construction industry from the perspective of managers. Understanding the context is key when designing effective interventions [38]; therefore, the aim of this study was to explore the barriers and facilitators of help-offering behaviour among managers in the construction industry to inform the development of a gender-responsive suicide prevention programme for the Irish construction industry.

## 2. Materials and Methods

### 2.1. Participants and Procedure

The current study utilised focus groups as the preferred data collection method to explore managers’ perspectives on help-offering in the construction industry. In line with the constructivist approach, focus groups provide opportunities for the co-construction and exploration of meaning and social norms [39]. This approach was in line with the study objectives and was deemed particularly appropriate for the male-dominated construction industry, where sociocultural factors have been strongly implicated in help-offering behaviours. Convenience sampling was used to recruit participants by leveraging existing connections with the companies involved with the wider project. The participants were eligible to take part in the study if they were over 18 years of age and employed as a manager in the construction industry. Within the context of the construction industry, the term ‘manager’ included health and safety staff; occupational health staff; supervisors; site foremen; and any other employees who had a responsibility for the health and wellbeing of construction workers more broadly. Five focus groups, comprising 33 participants, were conducted between January and September 2023. All the participants were internal managers employed with construction companies in Ireland. The sample consisted of Environmental Health and Safety staff (*n* = 19); project managers (*n* = 5); site managers (*n* = 2); a site foreman (*n* = 1); a medic (*n* = 1); contract managers (*n* = 2); site security *(n* = 1); a quantity Surveyor (*n* = 1); and planner/scheduler (*n* = 1). The mean age of the sample was 44.8 years (SD = 11.05), and the number of employees managed by the participants ranged from 8 to 500. The participants’ years of experience working in the construction industry ranged from 1 to 40 years. Some 76% of the participants (*n* = 25) had previously received formal mental health training, while 24% (*n* = 8) had not.

The key gatekeepers in each company were identified and contacted by the lead author, who explained this study’s purpose and requested their assistance with recruitment. Study information sheets were provided to the potential participants, and the lead author was available to answer any questions prior to the focus group. The focus groups were facilitated by the lead author who has previous experience with qualitative data collection. Prior to the commencement of the focus groups, the lead author reiterated the purpose of the study, and informed consent was obtained. The focus groups lasted approximately one hour. A topic guide was developed that explored the managers’ past experiences of having engaged with workers in distress. The topic guide was developed based on previous research and in consultation with the research team and was refined through an iterative process in response to the emerging findings from the focus groups. Semi-structured questions focused on the managers’ experiences of and reflections on the engagement process when supporting workers’ mental health and the stressors or challenges they faced when offering help to workers. This study formed part of a wider co-design process that was adopted for training development and engaging industry representatives as equal partners throughout the development process. Ethical approval for the study was granted by the South East Technological University’s Ethics Committee (Ethical Application Number 318).

### 2.2. Data Analysis

The focus groups were recorded and transcribed verbatim, with any identifying information removed. Reflexive thematic analysis (RTA) was used to analyse the data [40]. Analysis was conducted through six stages: (i) the familiarisation of data; (ii) initial coding; (iii) searching for themes; (iv) reviewing the themes; (v) defining the themes; and (vi) the writing up of the themes. Informal notes, focus group reports, and a reflective journal were kept by the lead author during the data collection process. Following completion of all the focus groups, the lead author read and re-read the transcripts to ensure familiarisation with the data. The data were manually coded line by line using Microsoft word and with reference to the informal notes taken by the lead author during and immediately after data collection. Manually coding allowed for full immersion with the data, in line with the principles of RTA. Initially, two transcripts were coded by each of the researchers to facilitate discussions and to explore emerging meaning in the data. This process of reflexively engaging with the data did not strive to reach consensus, but rather to explore the initial interpretations of patterns within the data.

From these discussions, a set of initial codes was formed. Initial coding was guided by the research question, and both semantic (surface-level) and latent (underlying) meanings were explored. However, as analysis progressed, the research team met regularly to discuss the meaning and patterns that were emerging across the codes. Analysis therefore was not bound by a specific coding framework, but rather reflected a more organic and subjective process of interpreting and making meaning out of each transcript. It followed an iterative process, whereby the researcher continuously modified analysis based on the codes generated in each transcript. Once all the transcripts were coded, the researcher re-read the transcripts again to gauge if the initial findings could be interpreted differently based on the latter phase of analysis. These codes were discussed with the research team to prompt deeper exploration or alternative interpretations of the data. Following this, similar codes were grouped together in a table in a word document, and subsequently grouped into preliminary themes.

The authors discussed and refined the codes and preliminary themes to ensure a meaningful and rich interpretation of the data, resulting in the final iteration of themes. The refined themes were linked with codes and quotes from the transcripts in a table in a word document to ensure that themes were consistent and representative of the meaning and patterns in the data. While positivist approaches to thematic analysis tend to rely on ‘data saturation’—defined as the point in which no new themes or codes emerge—reflexive thematic analysis acknowledges the researchers’ active role in interpreting and generating knowledge through an evolving and iterative process. Therefore, rather than seeking a specific sample size or fixed data saturation point, data collection ceased when the authors deemed that a sufficiently rich and nuanced interpretation of the data had been achieved in relation to the research question.

## 3. Results

Three themes were identified within the data. (i) Theme 1: creating the right environment; (ii) Theme 2: navigating the practice of help-offering; and (iii) Theme 3: using the right tools for the job. Themes provide a narrative of the many dynamic and interacting components that contribute to engagement and the help-offering process between managers and construction workers.

### 3.1. Creating the Right Environment

This theme encapsulates two sub-themes: (i) ‘fostering an open culture around mental health on-site’ and (ii) ‘overcoming systemic challenges’.

#### 3.1.1. Fostering an Open Culture Around Mental Health On-Site

Laying the groundwork to promote conversations around mental health was seen as being rooted in a broader workplace culture that promoted a supportive environment for the disclosure of mental health issues. Cultivating such a culture, however, faces a considerable obstacle in the form of mental health stigma, with many companies remaining ambivalent towards promoting the key messages around mental health:

“The stigma of mental health, no one wants to be talking about mental health, we know a lot of organisations that don’t have that openness of it’s okay to not be okay as we say”.

(FG403, Environmental Health and Safety Manager.)

Positioning oneself in a place to support a worker during a mental health crisis was not seen as something that happened overnight, but rather required collective and sustained efforts of building relationships and laying the groundwork for future engagement. Such efforts needed to ripple through the entire supply chain, ranging from the overarching workplace culture to the continued commitment of individual managers. At the site level, genuine and ongoing interactions between the managers and employees were seen as fundamental to building a culture that normalised conversations around mental health to enhance future engagement:

“Talking to them, just being friendly. You have to keep doing it. It’s constant. You have to make it normal, that’s what we do”.

(FG302, Project Manager.)

On a broader scale, this culture was seen as being cultivated by a company’s commitment to employee wellbeing. The provision of high-quality working conditions that demonstrated a genuine duty of care to employees was seen as a key enabler of engagement and as being instrumental to shaping employees’ willingness to disclose mental health issues. Conversely, the absence of such a commitment was seen as having a negative bearing on employee wellbeing:

“The facilities on-site are actually reasonable, like if the toilets actually work… what you’re showing is that you believe in people’s dignity working on-site. That actually leans into it, it doesn’t necessarily lead to positive mental health, but the opposite definitely leads into negative mental health”.

(FG102, Health and Safety Officer.)

While acknowledging that the overall commitment to employee wellbeing rested with the company owners/directors, the findings revealed that strong leadership was key when operationalising this commitment on the ground. The leaders who actively promoted and modelled engagement with wellbeing initiatives and mental health supports were seen as providing a catalyst for creating a collective mindset that prioritised mental health on-site and made workers feel safe to engage with support services:

“It’s down to creating that kind of, almost permission that it’s okay to go for a run or you know, go out to that organised activity”.

(FG303, Health and Safety Manager.)

“And that’s what we need to do, from a leadership point of view, to build that sense of everyone saying ‘let’s look after ourselves’ and knowing the company will help us out and take care of them. We can bring it into the supply chain from there”.

(FG302, Project Manager.)

#### 3.1.2. Overcoming Systemic Challenges

Despite strong support in principle for a more open and supportive environment around mental health and help-offering on-site, it was acknowledged that such aspirations did not always materialise in practice. The efforts to create this environment were often fraught with systemic challenges within the industry, with unrealistic deadlines and pressures from developers cited as the key barriers. The participants described being consistently torn between promoting employees’ mental health, whilst simultaneously ensuring they met the demands expected of them within their day-to-day professional role. For some, this created ambivalence as they sought to reconcile their desire to support workers’ mental health with concerns that by doing so, they faced potential backlash from senior management:

“The scale of the projects, it’s getting faster, and our clients want them built faster, once that pressure comes from the client to ourselves who would be a main contractor, our supply chain underneath us are feeling that pinch”.

(FG403, Environmental Health and Safety Manager.)

“If you’re going to let someone, take for example the crane driver, you let him go off, everything stops. And then you’re pushing harder when he comes back, because you have a deadline to meet, so you’ve lost that time, that time doesn’t reappear anywhere, so you’re putting more pressure on people to meet stupid dates”.

(FG501, Project Manager.)

Creating a workplace environment that was conducive to positive mental health was complicated further by the practice of sub-contracting within the industry. Sub-contractors’ more transient work practices stymied the site managers’ efforts at building relationships with them, resulting in ambivalence on the part of many sub-contractors towards buying into mental health initiatives and supports. This emerged as a particular challenge among the smaller and typically less well-resourced companies, often bound to tight profit margins and rigid project timelines:

“It’s up to the company to give the people the time over to be trained, and smaller companies wouldn’t do that. They would see that as a cost, they wouldn’t see that training as free”.

(FG501, Project Manager.)

Additionally, navigating different cultural attitudes and language barriers on-site posed an additional obstacle in engaging with foreign national workers, further hindering the participants’ efforts to promote a unified working environment. The participants spoke of a form of tribalism, whereby different nationalities tended to group together and to disengage from the broader site supports and community, thereby becoming a particularly hard-to-reach group:

“There’s a lot of foreign nationals working on this site… a lot of Irish working on this site and I find sometimes they don’t mix as well as we would hope”.

(FG205, Security Manager.)

“There’s a large number of the foreign guys who don’t speak English, you’re relying on an interpreter. I can’t see anyone with mental illness talking to you through an interpreter”.

(FG501, Project Manager.)

Moreover, addressing more restrictive and deeply engrained cultural norms that equated seeking help for mental health issues with weakness was particularly problematic with these groups. Thus, the managers faced an up-hill battle in terms of channelling their energies into repeatedly confronting this more systemic and traditional “macho” culture within the construction industry in order to overcome mental health stigma:

“In some cultures, when you start talking it means you’re whining. It means you’re chicken, they tend to use all those words, so you just have to toughen up and put that fence up and shoulders out”.

(FG206, Health and Safety Officer.)

Notwithstanding the valiant efforts by the individual managers on the ground, there was broad consensus that overcoming these systemic challenges to help-offering on-site must be through a co-ordinated, whole-system approach across the industry. Creating an environment that normalised mental health discussions and encouraged disclosure, was seen as being contingent upon endorsement at the senior leadership level and underpinned by legislation:

“I think the only way you get every contractor to buy into this is if you make it part of legislation that companies need to have a mental health leader”.

(FG501, Project Manager.)

### 3.2. Navigating the Practice of Help-Offering

Theme two outlines the key challenges faced by the managers as they navigated the many uncertainties and incongruities pertaining to the practice of help-offering.

There was broad consensus that workers who felt distressed were unlikely to take the initiative and approach a manager for help, but rather the onus lay on managers to intervene having been prompted to do so by a concerned colleague or having noticed the warning signs themselves. The participants noted their own vulnerability in offering help. They were particularly fearful about engaging with workers for the first time, not knowing how they were likely to respond, lacking confidence in their ability to intervene ‘correctly’, and potentially having to navigate a hostile response. Once again, these fears were compounded by the backdrop of a persistent ‘macho’, male-dominated environment that was ideologically opposed to expressing any kind of vulnerability. This left managers having to carefully assess the cost/benefit of approaching and engaging with a worker for the first time:

“I wasn’t very confident to be honest, I had one guy who would get very agitated, eventually I got to know him and understand him, but that first day, it really frightened me. It’s a scary place at the beginning, I won’t deny it. It’s completely intimidating, even still when I meet somebody new and if they are agitated it will frighten me, because you don’t know them, you know?”.

(FG402, Environmental Health and Safety Manager.)

“It’s very, very sensitive. Because if you go over to a guy and say ‘come here, are you alright?’ especially in construction, back to the macho thing, in two minutes you will be told where to go, who do you think you are? And I think in another case, a guy might actually open up to you. But I think we’re in a situation where it’s not as common as people would like it to be, where you can talk to people, you can approach people and say are you okay?”.

(FG101, Safety Advisor.)

A key influence on help-offering was having clear boundaries. The participants grappled with what mandated them to intervene and what the scope and extent of such an intervention should be. There was a fear of saying “the wrong thing” or “overstepping the mark” by impinging on workers’ privacy, along with feeling out of one’s depth and without the right ‘qualifications’ to offer help:

“And I think sometimes you need to be very, very careful because we are touching on a subject now where I certainly am not qualified or educated. I wouldn’t say qualified. I’m not educated in it enough, and I think before we start talking about this, you need to be sure that you don’t overstep your mark. You can certainly speak about it and talk about it and get a conversation going. But I was very aware that if I done the wrong thing…”

(FG101, Safety Advisor)

These concerns led to the preference for a more cautious and conservative approach to help-offering that accounted for the participants’ perceived limitations and provided clear boundaries, including separation from the role of health professional. Indeed, being clear about such boundaries was seen as pivotal in knowing when to disengage and refer a worker for more specialised support:

“And for some mental health issues, you know, it goes beyond having a chat over a cup of tea, if this person is unwell, they need to be seen by a medical professional”.

(FG203, Medic.)

“I was probably getting too involved at the very start years ago, whereas now I have my boundaries. I learned the hard way of getting too involved where I had a guy and [pause] we were searching [for him] for 2 days on one of our projects. I learned taking a step back is the most important thing and saying okay, I know my boundaries, that’s very, very crucial”.

(FG403, Environmental Health and Safety Manager.)

The practice of help-offering was complicated further by discrepancies across the companies regarding access to support services, with some only providing support services to direct employees, while others extended supports to all workers on-site, including those employed on a sub-contractual basis. This was particularly evident when managers engaged with a worker in distress who was not a direct employee of their company, leading to ‘grey areas’ and uncertainty about the duty-of-care boundaries in the absence of adequate support or guidance:

“They’re not an actual direct employee to you. Then what does that fall into? Should he be talking with someone within his company? Do you know what I’m saying, like…. It’s a grey area”.

(FG102, Health and Safety Officer.)

“Our company floats the supports to anybody that’s got our sticker on the helmet. I couldn’t stand up in front of all those hundreds of people and say we’ll mind you, but we won’t mind you”.

(FG201, Health Safety and Environment Manager.)

The participants highlighted the importance of maintaining a realistic perspective on what help-offering could achieve. While generally positively disposed to offering help, the participants remained acutely aware of the limitations when doing so. They were determined to ensure that a willingness to offer help was not seen as a panacea for addressing all of the complexities and challenges within the industry and the various personal, professional, and sociocultural factors affecting workers’ mental health. There was acknowledgement that even their best efforts in offering help could not address the root causes of many mental health issues:

“We can do nothing, we are powerless and there is no training in the world that is going to change if he is financially struggling, and there is no company in the world saying we are going to back him all the way. Very rarely”.

(FG101, Safety Advisor.)

As highlighted under theme one, the managers were cognizant of the need to navigate the practice of help-offering within the real world of meeting industry targets and maintaining the professional role expected of them. Despite valiant efforts at promoting positive engagement, the managers operated within a work environment that was challenging to all workers’ wellbeing, including themselves:

“We’re probably as guilty as anyone, you’re coming to a deadline and you’ve lads working around the clock like Saturdays, Sundays, full shifts, working all week without a break”.

(FG502, Project Manager.)

“It’s [long and arduous working conditions] the norm, so we know no better”.

(FG501, Project Manager.)

### 3.3. Using the Right ‘Tools for the Job’

This theme explores the crucial individual elements of the engagement process, encapsulating the key personal skills and qualities needed for effective help-offering.

Being open to conversations around mental health and presenting an empathetic and trustworthy demeanour were seen as the basis for initiating conversations on mental health and for offering help:

“It’s how you approach somebody is important, and being open to them, you know if they have a red flag in their head and they want to talk about it let them see that you’re a person they could ask a question”.

(FG305, Quantity Surveyor.)

Proficiency in the use of soft skills, such as empathy and active listening, were considered the key components in the engagement process. The findings highlighted that whilst managers tend to be highly trained in the professional or technical aspects of their role, they are less is invested in their interpersonal development. There was broad consensus that training courses were crucial in providing the managers with the right tools for engaging workers. There was, however, the perceived derogation of responsibility on the part of smaller contractors to provide such training, instead opting to piggy-back on the training provided by larger contractors which were seen as being better-resourced to do so:

“The larger sub-contractors would have invested money in it. The smaller guys don’t, they expect us to do that type of training with their guys and basically do that role for them”.

(FG501, Project Manager.)

The participants noted that willingness on their part to be vulnerable through the sharing of personal mental health challenges was an effective way to break the ice, build trust, and establish an open and safe space for employees in distress to reciprocate:

“Something that also helps, on my side at least, is going first. If you share something vulnerable about yourself, or go first, it sort of encourages the rest of the conversation, which is mostly with men”.

(FG306, Project Manager.)

“We go for tea or coffee, and I would always start, and before they tell their story, I’ll tell my story very, very briefly and they open up to me straight away”.

(FG403, Environmental Health and Safety Manager.)

Additionally, a shift away from the more traditional authoritarian management styles that were seen as having alienated employees in the past was highlighted as a further step towards promoting engagement between workers and the managers. Whilst it was acknowledged that this is still a work in progress, there were numerous references to ongoing efforts to promote a more positive, team-based environment that fostered meaningful relationships between the managers and employees:

“I grew up in an era where a lot of senior management as well as our site agents at the time were tyrants, like you wouldn’t be going near them, and you know that approach has changed big time in the last 10–15 years in the industry”.

(FG304, Contracts Manager.)

This approachability was considered the key driver of engagement and paved the way for positive relationships that, in turn, fostered a sense of comradery between the managers and workers. The potential for engaging with workers through these informal interactions was seen as integral to building rapport and creating a safe space for employees to discuss mental health:

“A manager now, you can have a conversation with them, go for a cup of coffee with them. 15–20 years ago that wouldn’t have happened”.

(FG304, Contracts Manager.)

“We have what we used to say was the craic with the lads, but if you are one of those guys who think they are the lord almighty himself. Nobody will talk to you”.

(FG101, Safety Advisor.)

Additionally, allowing for time to build genuine relationships and to get to know workers on a personal level acted as a natural and less-daunting bridge to any potential subsequent conversations about mental health:

“If someone comes to you with a problem and then you can tell them what their wife’s name is, or if they’ve got kids, if you can make it personal, they’ll trust you a bit more because they feel as if you know them, whereas, some people would barely even know your name on a team you know or anything about you”.

(FG305, Quantity Surveyor.)

This ‘personal touch’ was not seen as something that ‘just happened’ organically, but rather required a sustained effort by managers to build their knowledge of a worker’s normal or baseline demeanour or behaviour, which could then serve as a method of identifying warning ‘signs’. This approach was highlighted as one of the most significant enablers of help-offering:

“I was very familiar with the type of person he was and I started to see him becoming kind of bit more reclusive. He wouldn’t talk to people, so I would always then just, you know, every time I see him start up a conversation. And we’d talk about anything but then gradually over time he was opening up to me and then kind of later on down the line when he found things hard, he was actually ringing me and I was talking to him then”.

(FG405, Environmental Health and Safety Advisor.)

Several impediments emerged in terms of applying these tools for the job in practice. The most notable of these was the transient nature of the work which gave rise to short-term contracts (see Section 3.1.2), making sustained contact with workers more challenging:

“When you know a person, it can be easier. It can be hard on the projects where it might be a new project and you mightn’t be familiar with the people, it takes some time I think, to develop a rapport with someone so that you might notice changes in their behaviour”.

(FG405, Environmental Health and Safety Advisor.)

Furthermore, the findings revealed that workers often delay seeking help or seek help covertly, fearing that the disclosure of mental health issues might have negative career ramifications or result in the workers being perceived as unreliable. The managers’ ability to reassure workers of confidentiality throughout the engagement process was seen as crucial for promoting disclosure and help-seeking among workers:

“I would have a lot of people coming to me and they’re like can you not report this to anybody and this type of attitude. I say ‘you’re here with me, it’s completely confidential unless it is a medical emergency’, we need to maintain that barrier of confidentiality”.

(FG203, Medic.)

## 4. Discussion

The aim of this study was to explore the key barriers and enabling factors that influence managers’ capacity and willingness to offer help to construction workers in distress. By highlighting the nuanced and multi-faceted nature of help-offering behaviour and the sociocultural context of the construction setting, this study sheds light on the unique position of managers to offer help to workers. Additionally, the findings provide a roadmap for future intervention design and have directly informed the development and pilot delivery of a suicide prevention programme for the Irish construction industry. The Social–Ecological Model [6] was chosen as an appropriate framework to understand the factors that influence help-offering behaviour, encompassing organisational factors (policies and leadership), individual factors (knowledge, skills, and attitudes), intrapersonal factors (relationships and peer influence), and community factors (industry norms).

At the organisational level, there was broad consensus that the wider normative culture around mental health on-site was the crucial precursor to help-offering. Fostering such a workplace culture that normalises conversations about mental health, challenges stigma, and works towards transcending the traditional ‘macho’ environment within the construction industry was seen as facilitating the conditions most conducive to individual help-offering. The findings highlight the need for a shift away from viewing men as “the problem”—emotionally inept and resistant to seeking help [41]—to working on creating a safe space conducive to help-offering and the disclosure of mental health issues. Importantly, engaging with and supporting workers in seeking help must be grounded in authenticity and non-tokenistic efforts [42], as demonstrated through wider company commitment, ethos, and genuine care for employees. Creating such a culture was seen as an ongoing process, requiring a whole-system approach that cultivated positive attitudes towards mental health throughout the supply chain. The findings complement existing best-practice research, which recommends that establishing a culture of health in the workplace requires a multi-faceted approach, encompassing buy-in from senior management, supportive middle management, and peer support [43]. This collective approach has been shown to be the catalyst for wider culture change and increased openness towards mental health within the construction industry [44].

Several challenges emerged, however, in the pursuit of this cultural holy grail. These included inconsistencies across the companies regarding attitudes towards mental health, the varying degrees of importance placed on promoting mental health initiatives on-site, the complexities of navigating relationships between main and sub-contractors, as well as the difficulties in managing diverse cultural attitudes towards mental health and language barriers on-site. The reliance on sub-contracting in the industry posed a significant barrier to engagement, with sub-contractors often operating within their own micro-cultures and practices [45]. Smaller contractors were highlighted as being particularly difficult to engage in health promotion activities, reinforcing existing research that health promotion is a lower priority for small-to-medium companies in the construction sector [46]. Additionally, the participants were uncertain about who was responsible for health promotion and help-offering across multiple companies on-site, aligning with the existing findings that inconsistent mental health approaches and unclear responsibilities around duty of care are common across the industry [47]. Moreover, language barriers and disengagement from foreign workers further complicated the process [48].

Whilst mental health promotion at the organisational level is crucial for lasting culture change, organisational-level interventions have been found to be more effective when combined with complementary individual-level approaches. Generally, individual-level interventions have a stronger impact on individual behaviour and mental health outcomes [12,24] and underscore the significant role managers play in supporting workers within the broader context of cultural and organisational changes. The findings highlight that willingness to offer help was influenced by ambiguity around the accountability for workers’ wellbeing, personal skill deficits, and uncertainties around professional boundaries. The participants noted fear around ‘overstepping the mark’ and initiating conversations around mental health that might not be acceptable to workers. This reinforces the previous findings that those in managerial roles often lack clarity around addressing mental health issues in the workplace [29]. Additionally, the participants identified time as a key barrier to help-offering, with pressures from senior management restricting their capacity to engage with workers. In line with the existing findings, health-promotion activities are sometimes seen as hindering productivity, especially when working to tight deadlines [45]. However, the participants highlighted that managers who are willing to model mental health promotion can challenge this view and encourage engagement, emphasising the importance of the top-down endorsement of health promotion in the workplace [49].

At the intrapersonal level, reciprocity, trust, and relationship building were identified as the key enablers to help-offering in the industry. Despite the presence of multiple companies, workers, and agencies on-site at any given time, the participants stressed the importance of promoting a unified working environment and a sense of comradery on-site. Building these connections with workers was seen as paving the way for future help-offering opportunities, with the participants noting that ‘walking the site’ and getting to know workers was an important engagement strategy. This hands-on approach and less-hierarchical leadership style have previously been found to appeal to workers in the construction industry [48]. Leaders willing to get out on-site can leverage the idea of men working ‘shoulder to shoulder’ in a meaningful activity of work, while simultaneously providing an opportunity for men to discuss mental health in an informal, familiar space [50]. Additionally, men are more likely to seek help when they perceive a reciprocal exchange [51], and the participants noted that willingness to share personal stories of mental health challenges can create permission and affirmation for workers to disclose mental health issues in a safe environment [52].

The findings revealed that construction workers rarely approached others for help but rather relied on peers and/or managers to recognise warning signs and deviations from their usual demeanour. This is an important finding, as men often rely on significant others in trusting communities to prompt help-seeking [32,53]. This underscores the importance of health promotion initiatives that train gatekeepers in the construction industry to foster a supportive environment for disclosing mental health issues. At the community level, these interpersonal interactions are foundational for shaping the broader workplace culture that dismantles restrictive masculine norms, often encompassing a focus on self-reliance, toughness [54], and a ‘man-up’ culture [55]. Consistent with previous research, the findings suggest that a willingness to model vulnerability works towards dismantling stigma in an informal setting that may be more acceptable to men [56]. Work is ongoing in the industry to rework more traditional masculine norms through informal, peer-led conversations, where vulnerability is remodelled as strength to encourage a culture of comradery and looking out for one another [50].

### 4.1. Implications for Practice and Future Research

Managers play a key role in workplace health promotion. However, the findings highlight the need for a more integrated approach to workplace health promotion in the construction industry that supports individual managers in their role of help-offering, while working in tandem with broader organisational efforts [25]. Indeed, individual-level approaches alone are critiqued for drawing attention away from the wider psychosocial working environment and culture [57]. Future efforts should avoid over-emphasising individual workers’ responsibility [58], and instead view individual help-offering as just one cog in the wheel, working in conjunction with wider industry approaches to build a positive culture around mental health. This can be best facilitated through the development of policies and procedures that establish industry-wide standards around promoting mental health and creating a psychologically safe workplace. Additionally, it is recommended that training on psychosocial risks be delivered to all stakeholders in the industry, with workers involved in the co-design of training resources and workload reviews [59].

Moreover, more work is needed to address systemic challenges, such as unrealistic deadlines, sub-contractor management, and maintaining connections with workers in the context of a transient industry. These findings align with the existing calls for industry-wide approaches to ensure all workers have equal access to health-promotion activities [45], regardless of their contractual working arrangements. Managers’ ability to support workers will remain limited unless wider systemic challenges, such as unrealistic deadlines from developers, are addressed. More consistent top-down buy-in is needed to drive long-term change [60]. It is also recommended that future research engage with a wider range of stakeholders to explore strategies to address the wider psychosocial risks to workers’ wellbeing alongside the ongoing efforts of managers on-site. This aligns with existing research that calls for government intervention in the financial infrastructure of the industry, along with macro-level solutions, such as limiting overtime hours and offering greater worker flexibility [61]

Managers, nevertheless, continue to play a key role in shaping employee attitudes towards mental health promotion at the site level. It is recommended that industry-wide guidelines for engaging construction workers around their mental health are developed. These guidelines should address communication barriers and establish clear protocols in relation to help-offering and dealing with disclosure at the site level. Additionally, they should include a clear, standardised support pathway that is freely available to all workers on-site. This approach would alleviate managers’ uncertainty when navigating and engaging with workers employed on a sub-contractual basis. Future manager training should be tailored to the unique needs of the industry, focusing on soft skill development to build confidence to support workers, while clarifying the limitations of help-offering and assisting managers in balancing demands [62]. Furthermore, future training should account for the influence of traditional masculine norms and utilise the existing frameworks for designing men’s health interventions to ensure the acceptability of training programmes [63]. A co-production approach is recommended for designing such training, particularly in male-dominated industries, where engaging men as equal partners throughout the process leads to more acceptable training and engagement [64].

Further research is needed to explore the experiences of managers in small-to-medium-sized enterprises, drawing from a more diverse geographical sample. The experiences of managers in the main contracting companies may differ from those in smaller companies, and the needs for training may vary accordingly. Moreover, future studies should investigate the cultural and language barriers from the perspective of workers from diverse nationalities on-site. This would allow for the development of a culturally sensitive manager training that meets the needs of workers from diverse backgrounds on-site. In conjunction with ongoing suicide prevention efforts at the individual level, more research is needed with a wider range of stakeholders in the industry to further examine the impact of the wider psychosocial risks to construction workers’ mental health and to co-produce potential solutions.

### 4.2. Limitations

There are several limitations to consider when interpreting the current findings. This study engaged only with construction companies already involved in the wider project. Thus, all the participants were employed by larger contractors, all of whom have access to more resources and supports compared to those of smaller contractors. In 2020, small-to-medium-sized enterprises accounted for 91.6% of the persons employed in the construction industry in Ireland [65]. Therefore, the results of the current study may not be generalisable to the construction industry more broadly as they primarily reflect the experiences of those employed by larger contractors. Future research should aim to recruit managers from smaller companies to ensure the more balanced representation of the construction sector in Ireland. Furthermore, most participants in this study had previously received mental health training and were likely to have a particular interest in construction workers’ mental health. They may not, therefore, accurately reflect the views of all managers within the industry. Additionally, while a qualitative approach was deemed to be best suited to meeting the objectives of the current study, reflexive thematic analysis relies on the researcher’s active interpretation and generation of the findings. Therefore, there is a potential for bias. While the Social–Ecological Model of health and Connell’s relational theory of masculinities provide a nuanced understanding of the dynamics of help-offering, these theories are not sufficient to explore motivation or measurable intention to offer help. While the current study is a starting point, future research might utilise other theories, such as the theory of planned behaviour [66], to further investigate the components that influence the intention to engage with workers.

## 5. Conclusions

This study explored the key barriers and enabling factors that influence managers’ capacity or willingness to offer help to construction workers in distress. The findings offer important insights into the multi-faceted nature of the engagement process in male-dominated industries and provide guidance for future intervention development efforts. Broadly, the intention to offer help was influenced by the wider workplace culture encompassing the acceptability of engaging with workers, top-down management support, wider systemic challenges, and a whole-system approach to health promotion that is consistent across the supply chain. The findings indicate that managers would benefit from gender-responsive gatekeeper training tailored to the context of the industry that accounts for gender norms and expectations and engagement approaches that men find more acceptable.

## Data Availability

The data underlying this article cannot be shared publicly due to privacy and ethical considerations related to the sensitive nature of the study. However, the data will be shared upon reasonable request to the corresponding author.
